# Therminator DNA Polymerase: Modified Nucleotides and Unnatural Substrates

**DOI:** 10.3389/fmolb.2019.00028

**Published:** 2019-04-24

**Authors:** Andrew F. Gardner, Kiserian M. Jackson, Madeleine M. Boyle, Jackson A. Buss, Vladimir Potapov, Alexandra M. Gehring, Kelly M. Zatopek, Ivan R. Corrêa Jr., Jennifer L. Ong, William E. Jack

**Affiliations:** New England Biolabs, Inc., Ipswich, MA, United States

**Keywords:** DNA sequencing, xenonucleic acids, aptamers, functionalized DNA, unnatural base pairs

## Abstract

A variant of 9°N DNA polymerase [Genbank ID (AAA88769.1)] with three mutations (D141A, E143A, A485L) and commercialized under the name “Therminator DNA polymerase” has the ability to incorporate a variety of modified nucleotide classes. This Review focuses on how Therminator DNA Polymerase has enabled new technologies in synthetic biology and DNA sequencing. In addition, we discuss mechanisms for increased modified nucleotide incorporation.

DNA polymerases have evolved to efficiently and faithfully replicate DNA. To maintain replication fidelity, DNA polymerases have evolved mechanisms for exquisite selectivity to insert the correct nucleotide across its complementary templating base. In all families of DNA polymerases, DNA polymerization occurs via a well-studied generalized mechanism for activity (Steitz, 1999). After DNA template binding, a DNA polymerase binds a correct deoxynucleotide (dNTP) to induce a slow rate-limiting step. This slow step is proposed to represent a conformational change from an open to a closed state, positioning the enzyme active site, DNA template and dNTP in the correct conformation for catalysis. A rapid chemical reaction catalyzes phosphoryl transfer of the bound dNTP to the DNA template and produces one inorganic pyrophosphate. Finally, the closed state reverts back to an open relaxed state, inorganic pyrophosphate is released and the complex either dissociates or translocates along the DNA, poised to add the next nucleotide (Xia and Konigsberg, 2014).

Family A and Family B DNA polymerases active sites are arranged to stabilize the correct incoming dNTP in the proper geometry for catalysis [Reviewed in (Steitz, 1999)]. The dNTP deoxyribose moiety assumes a favorable 3′-endo-sugar conformation. This conformation is constrained by hydrogen bonds between the 3′-OH and a main chain amide (corresponding to Vent DNA Polymerase position 412) and a non-bridging β-phosphate oxygen. Nucleotide α, β, and γ-phosphates are further stabilized by direct or water-mediated hydrogen bonds with active site residues (Gardner and Jack, 2002). The absence of the 3′-OH on modified nucleotides (such as ddNTPs or 3′-*O*-azidomethyl-dNTPs) disrupts hydrogen bonding with the β-phosphate (and main chain amide), potentially increasing the activation energy required to orient the α-phosphate for phosphoryl transfer. Because of this intricate network of active site interactions between amino acid side chains and the correct dNTP, any structural modification to the nucleotide structure disrupts this active site network of interactions and leads to discrimination against unnatural nucleotides. Therefore, to efficiently incorporate modified nucleotides, the DNA polymerase active site needs to be engineered to accommodate a variety of nucleotide structural variants. This review examines one engineered DNA polymerase called Therminator DNA Polymerase and discusses recent applications and mechanisms for enhanced modified nucleotide incorporation.

## Therminator DNA Polymerase

**Figure 1:**
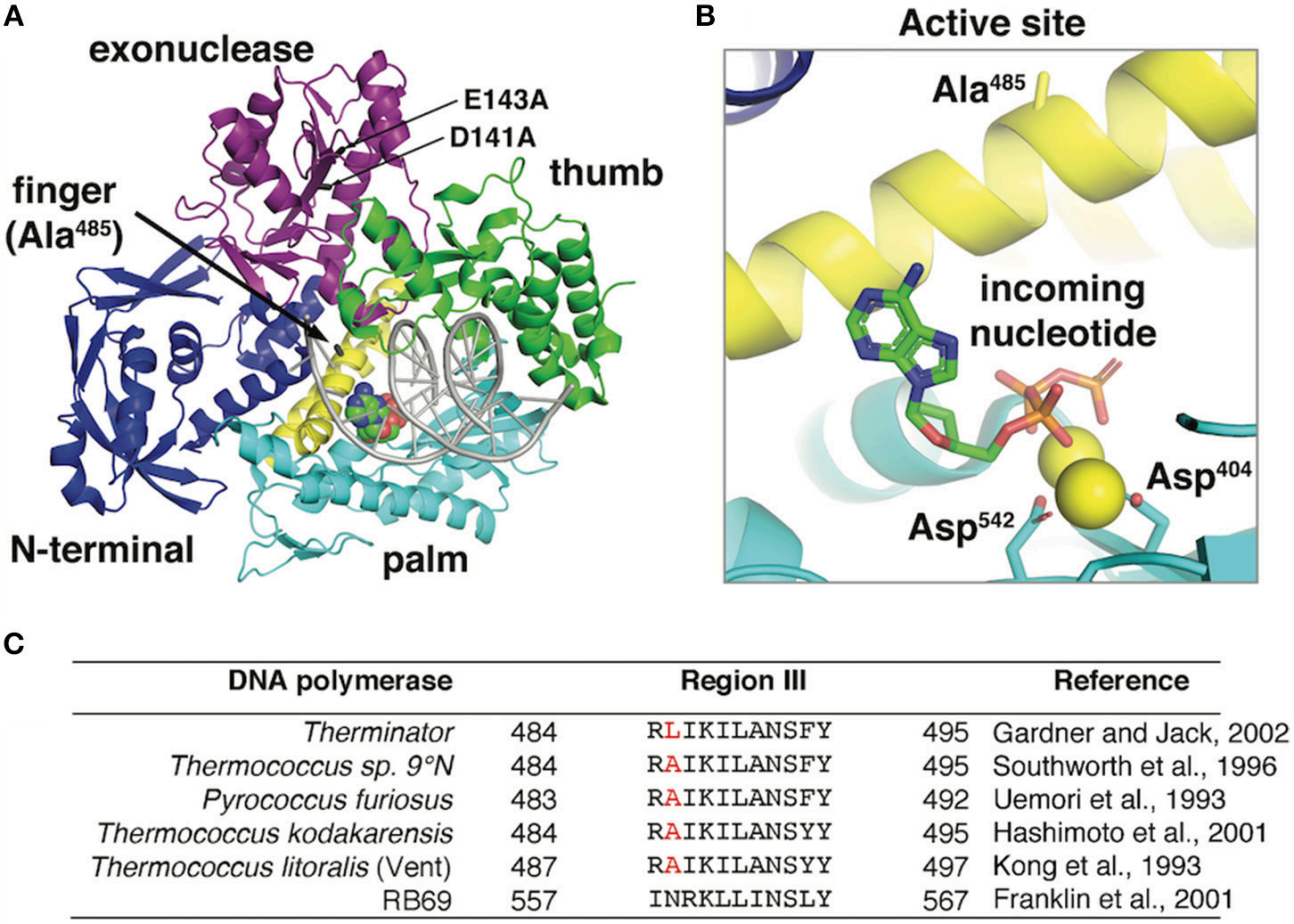
**(A)** The 9°N DNA polymerase exo- crystal structure [PDB ID: 5OMV (Bergen et al., 2013)] in complex with a primer:template (gray) and incoming nucleotide (spacefill) is shown. The position of D141A/E143A mutations in the exonuclease domain and the Therminator DNA Polymerase mutation (A485L) are highlighted. **(B)** The 9°N DNA polymerase active site shows the incoming nucleotide and catalytic aspartates (D404 and D542) stabilizing the nucleotide triphosphates. Alanine 485 in the 9°N DNA Polymerase exo- structure points away from the active site and does not directly interact with the incoming nucleotide. The Therminator DNA Polymerase mutation (A485L) increases the side chain size at position A485 but this structure has not yet been solved. **(C)** DNA polymerase Region III conserved active site residues in hyperthermophilic archaea and bacteriophage RB69 (Kong et al., 1993; Uemori et al., 1993; Southworth et al., 1996; Franklin et al., 2001; Hashimoto et al., 2001; Gardner and Jack, 2002) were aligned using Clustal Omega (Sievers and Higgins, 2018). Therminator DNA polymerase (D141A/E143A/A485L) is derived from the parental DNA polymerase from *Thermococcus* sp. 9°N (GenBank: AAA88769.1). The position of the Therminator DNA polymerase mutation (A485L) is highlighted in red.

Early studies using a DNA polymerase from *Thermococcus litoralis* (Vent DNA Polymerase exo-), demonstrated that mutating an active site alanine 488 to a larger, more bulky side chain increased the efficiency of modified nucleotides including ddNTPs, rNTPs, and 3′-dNTPs (Cordycepin) (Gardner and Jack, 1999). Similar increases in modified nucleotide incorporation efficiencies were demonstrated in related DNA polymerases by increasing the equivalent alanine position to larger amino acid side chains, Pfu exo-/A486Y (Evans et al., 2000); KOD exo-/A485L (Hoshino et al., 2016); Tgo exo-/A485L (Pinheiro et al., 2012)]. The Vent/A488L mutation was transferred to a similar exonuclease deficient (exo-) hyperthermophilic DNA polymerase from *Thermococcus* sp. 9°N resulting in the commercial Therminator DNA Polymerase. *Thermococcus* sp. 9°N is an anaerobic hyperthermophilic euryarchaeon isolated from scrapings of a deep sea volcanic smoker chimney collected at the 9°N East Pacific Rise vent site, 500 miles south of Acapulco, Mexico at a depth of 2500 meters (Southworth et al., 1996). Specifically, Therminator DNA Polymerase is derived from the Family B *Thermococcus* sp. 9°N DNA Polymerase (GenBank: AAA88769.1) (Southworth et al., 1996) and contains mutations in the conserved exonuclease domain (separate from the polymerase active site domain) (D141A/E143A) and a mutation (A485L) in the conserved polymerase active site Region III (Figure 1). The D141A/E143A mutations inactivate the 3′-5′ exonuclease activity so that any modified nucleotide that is incorporated is not subsequently removed by the 3′-5′ exonuclease proofreading activity.

### A Model for Therminator DNA Polymerase for Increased Modified Nucleotide Incorporation

Three-dimensional structural information provides limited clues as to the positioning and mechanism of the Therminator DNA Polymerase A485L mutation. In the parental 9°N DNA Polymerase crystal structure, alanine 485 is located on the O-helix Finger domain facing away from the incoming nucleotide in the active site [PDB ID: 5OMV (Rodriguez et al., 2000; Bergen et al., 2013; Kropp et al., 2017)] (Figure 1). Therefore, mutations at 485 will not directly contact the incoming nucleotide and likely acts indirectly by reducing steric barriers for nucleotide analog binding or facilitating a conformational change during polymerization. Alternatively, since the Finger domain undergoes a conformational change upon nucleotide binding, the A485L mutation may alter the equilibrium between an open and closed active site which may decrease discrimination for modified nucleotides. Kinetic data demonstrated that in Vent DNA Polymerase, the alanine to leucine mutation (A488L) modestly (~2-4-fold) increased both *k*_pol_ and modified nucleotide binding (*K*_D_). Unfortunately, the direct mechanism for reduced discrimination against modified nucleotides by Therminator DNA Polymerase is not completely understood. Additional research is needed to definitively determine how the mutation affects the conformational change rate and if modulation of the conformational change by the mutation is important for nucleotide analog discrimination.

## Applications of Therminator DNA Polymerase

**Figure 2:**
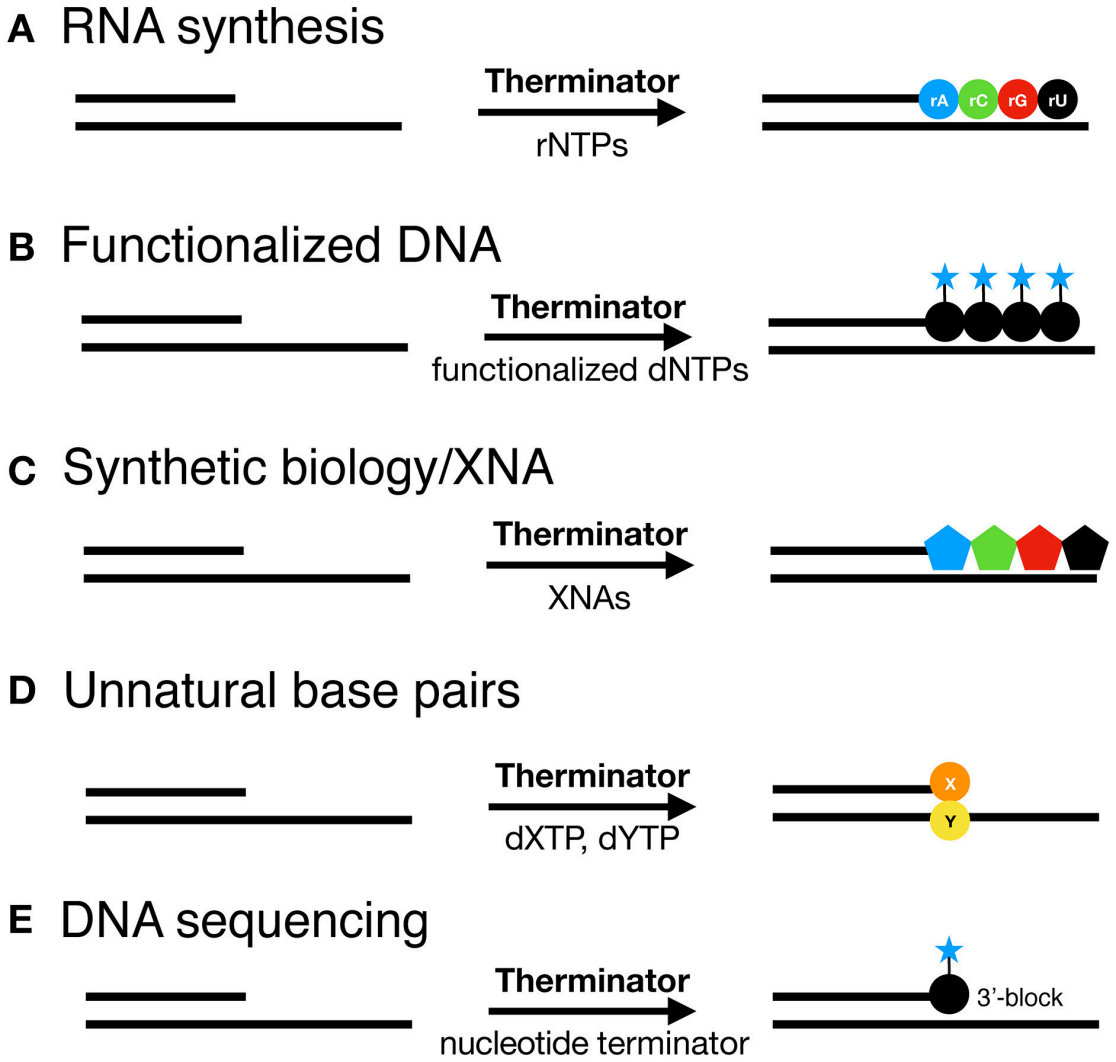
Therminator DNA Polymerase incorporates a variety of modified nucleotide to synthesize on a DNA template **(A)** RNA, **(B)** Functionalized DNA, **(C)** XNA for synthetic biology, **(D)** Unnatural base pairs and enables **(E)** DNA sequencing methods.

**Figure 3:**
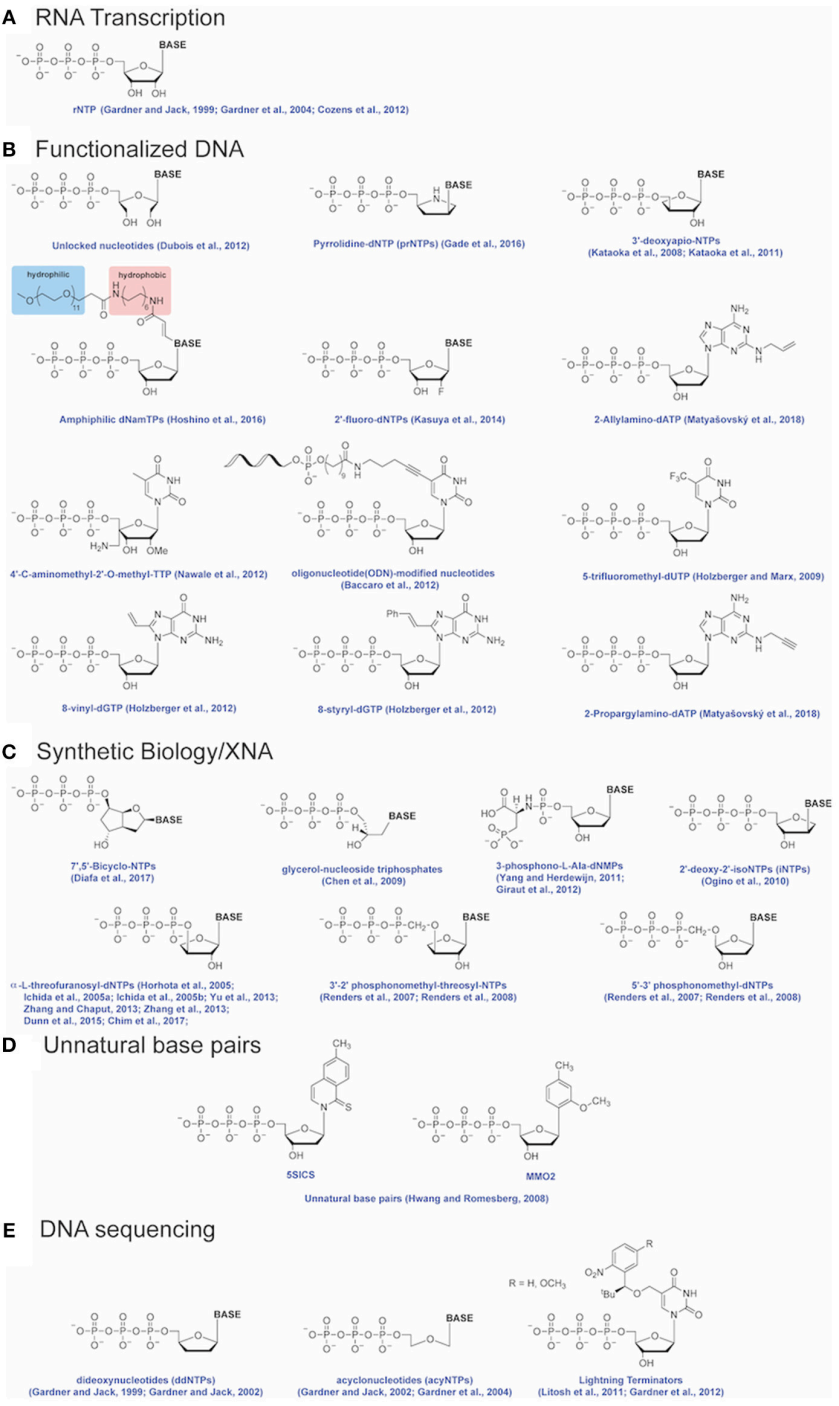
Modified nucleotides. Modified nucleotides incorporated by Therminator DNA Polymerase for applications including **(A)** RNA transcription, **(B)** Functionalized DNA, **(C)** Synthetic Biology/XNA, **(D)** Unnatural base pairs and **(E)** DNA sequencing. References for each modified nucleotide are provided.

Engineered DNA polymerases capable of synthesizing modified nucleic acids have enabled emerging and foundational technologies, including synthetic biology (Houlihan et al., 2017a,b), aptamer therapeutics (Lapa et al., 2016) and DNA sequencing (Slatko et al., 2018). This Review focuses on how Therminator DNA Polymerase has been used as a tool to label DNA, synthesize modified substrates, for example containing an expanded genetic alphabet, and has enabled DNA sequencing-by-synthesis and genotyping methodologies (schematically depicted in Figure 2). Incorporation of a variety of modified nucleotides by Therminator DNA polymerase depicted in Figure 3 will be discussed below.

## Synthesis of RNA Using Therminator DNA Polymerase

Ribonucleotide triphosphates (rNTPs, used for RNA synthesis) occur at a much higher concentration than deoxyriboncleotide triphosphates (dNTPs) inside cells (Williams and Kunkel, 2014). Therefore, DNA polymerases have evolved mechanisms to ensure selection of the correct nucleotide (dNTPs) in order to maintain the integrity of DNA. The DNA polymerase “steric gate” excludes rNTPs by a clash between bulky amino acids and the rNTP 2′-OH. Steric gate amino acids are well studied in Family A and B DNA polymerases (Brown and Suo, 2011). In 9°N DNA Polymerase, a Family B DNA polymerase, the conserved steric gate residue is Y409. Vent DNA Polymerase discriminates against rCTP incorporation via a 16-fold reduced binding affinity (*K*_D_ = 1100 μM) and a 400-fold slower rate of incorporation (*k*_pol_ = 0.160 s^−1^) compared to dCTP (Gardner et al., 2004). When Y409 is mutated to a smaller amino acid such as valine, up to five successive ribonucleotides can be incorporated (Gardner and Jack, 1999).

Therminator DNA Polymerase retains the wildtype Y409 steric gate amino acid. However, the A485L mutation reduces discrimination for rNTPs and allows incorporation of up to twenty ribonucleotides (Gardner and Jack, 1999; Gardner et al., 2004). In addition, the equivalent mutation in Vent DNA Polymerase (A488L) incorporated rCTP more efficiently than wild-type due to a higher binding affinity (*K*_D_ = 360 μM) and faster rate of incorporation (*k*_pol_ = 0.7 s^−1^) (Gardner et al., 2004). Presumably, RNA synthesis is limited to short products due to additional fidelity checkpoints and constraints that favor the synthesis of correct B-form DNA. Additional mutations engineered into the equivalent Therminator DNA Polymerase mutant (A485L) from *Thermococcus gorgonarius* (Tgo exo-/Y409G/A485L/E665K), dramatically increased ribonucleotide incorporation up to a 1.7 kb RNA product (Cozens et al., 2012). These additional mutations permit synthesis of A-form RNA:DNA molecules and may be used to create long RNAs.

## Synthesis of Labeled DNA Using Therminator DNA Polymerase

Many applications label DNA using modified nucleotides as detection reagents. Dye-derivatized nucleotides and analogs are commonly used for fluorescent detection during automated DNA sequencing (Prober et al., 1987), detection of single nucleotide polymorphisms (Chen and Kwok, 1999) and genome-wide mapping (Xiao et al., 2007). DNA polymerases from Families A and B have different preferences for labeled nucleotides based on linker arm length, dye charge and structure (Brandis, 1999; Gardner and Jack, 2002). For example, Therminator DNA Polymerase incorporates some dye-labeled nucleotides more efficiently than others suggesting that the dye structure can affect polymerase binding and incorporation. For example, modified nucleotides labeled with rhodamine dyes (TAMRA, ROX, R6G, and FL-12) are incorporated more efficiently than Cyanine-3 or Cyanine-5 dyes (Gardner and Jack, 2002). In addition to dye-labels, Therminator DNA Polymerase incorporates fluorescent analogs for use in fluorescence spectroscopy studies such as 8-vinyl-dGTP and 8-styryl-dGTP (Holzberger et al., 2012).

In addition to fluorescent labeling, nucleotides derivatized with a variety of reporter molecules act as important detection reagents. Therminator DNA Polymerase incorporates an amino functionalized analog [pyrrolidine ddNTP (prNTPs)] that enables conjugation without modifying the structure of the nucleobase (Gade et al., 2016). Matyasovky et al. designed base modified 2-allyl- and 2-proparylamino-dATP to introduce modifications into the minor groove of DNA via thiol-ene addition or CuAAC click chemistry (Matyašovsk et al., 2018). Another modified nucleotide designed by Marx and colleagues conjugated an oligonucleotide barcode to the base (ODNs) (Baccaro et al., 2012). Despite the very large base modification, Therminator DNA Polymerase synthesizes ODNs to form oligonucleotide barcoded substrates that could be used as hybridization probes for subsequent readout (Baccaro et al., 2012).

Synthesis of long modified DNAs may require high processivity yet Therminator DNA Polymerase is distributive, incorporating less than 20 nucleotides per binding event (Williams et al., 2008). To increase processivity and synthesize longer molecules, researchers attached two biotinylated peptide “legs” to Therminator DNA Polymerase and formed a complex with streptavidin beads. Then the modified Therminator DNA Polymerase-streptavidin bead complex bound DNA and improved processivity from less 20 nucleotides to several thousand nucleotides per binding event (Williams et al., 2008).

## Synthesis of Modified Functional Polymers and Aptamers Using Therminator DNA Polymerase

Therminator DNA Polymerase has been used to polymerize unnatural nucleotides to expand the functionality of DNA and alter its physical and chemical properties. Functionalized nucleic acids resistant to cellular nucleases may be used as aptamer therapeutics to inhibit protein targets (Lapa et al., 2016). Introducing chemical modifications of nucleotides increases the chemical diversity of synthesized polymers [reviewed in (Anosova et al., 2016)]. Hoshino and colleagues demonstrated incorporation of amphiphilic dNTP analogs by Therminator DNA Polymerase to create highly modified DNA molecules (Hoshino et al., 2016). Locked nucleic acids (LNAs) contain a ribofuranose ring in a locked, single conformation leading to duplex stabilization (Vester and Wengel, 2004). Alternatively, “unlocked” nucleic acids (UNAs) synthesized by Therminator DNA Polymerase offer additional substrate functionalization (Dubois et al., 2012). Other examples of functionalized DNAs made by Therminator DNA Polymerase using modified nucleotides include 2′-fluoro-NTPs (Kasuya et al., 2014), glyceronucleotides (gNTPs) (Chen et al., 2009), 7′,5′-Bicyclo-NTPs (Diafa et al., 2017), 3-phosphono-L-Ala-dNMPs (Yang and Herdewijn, 2011; Giraut et al., 2012), 3′-2′-phosphonomethyl-threosyl-NTPs (Renders et al., 2007, 2008), 5′-3′-phosphonomethyl-dNTPs (Renders et al., 2007, 2008), 2′-deoxy-2′-isonucleoside (iNTPs) (Ogino et al., 2010), 3′-deoxyapionucleotide 3′-triphosphates (apioNTPs) (Kataoka et al., 2008, 2011), 5-trifluoromethyl-dUTP (Holzberger and Marx, 2009) and 4′-C-aminomethyl-2′-O-methyl-TTP (Nawale et al., 2012). Kinetic studies of gNTP incorporation by Therminator DNA Polymerase demonstrate that while the rate of gNTP incorporation (*k*_cat_ = 0.8–4.7 s^−1^) is similar to dNTPs (*k*_cat_ = 2–4.2 s^−1^), gNTPs are bound with >350-fold lower affinity. For example, the *K*_m_ for TTP is 0.35 μM while the *K*_m_ for gTTP is 129 μM. Each of these modifications introduce functional diversity that may be useful for generating novel aptamers.

In addition to incorporating modified nucleotides on a natural DNA template, Therminator DNA Polymerase accommodates modifications in the template strand. For example, Xiao et al. demonstrate high fidelity synthesis of dATP across from mirror image L-thymidine in the template strand using Therminator DNA Polymerase (Xiao et al., 2017). Therefore, the ability of Therminator DNA Polymerase to synthesize modifications on both the primer and template strands will increase the diversity of modifications that can be introduced into substrates.

Xenonucleic acids [XNAs; reviewed in (Anosova et al., 2016)] are artificial genetic polymers that may be used to store genetic information in non-natural templates. New genetic scaffolds of modified nucleotides have the potential of building biosafe substrates for synthetic biology (Schmidt, 2010). Threose nucleic acid (TNAs: α-L-threofuanosyl-(3′-2′) nucleic acid) is an unnatural genetic polymer that is nuclease resistant (Horhota et al., 2005; Ichida et al., 2005a,b; Yu et al., 2013; Zhang and Chaput, 2013; Zhang et al., 2013; Dunn et al., 2015; Chim et al., 2017). In TNAs, the natural 5′-carbon ribose sugar is replaced with a four carbon threose sugar while phosphodiester bonds occur at the 2′ and 3′ positions. Threose has been found in pre-biotic reactions and on meteorites suggesting that the sugar can form spontaneously (Zhang and Chaput, 2013). Therminator DNA Polymerase can copy a 90 nucleotide DNA template into TNA using threose-NTPs (tNTPs) at a rate only four times slower than natural dNTPs (Dunn et al., 2015). For example, the observed rate (*k*_obs_) of dCTP incorporation is 4.2 s^−1^ compared to 0.9 s^−1^ for tCTP (Dunn et al., 2015). Incorporation of TNA with Therminator DNA Polymerase enables further study of the structure and function of TNAs. Continued engineering of Therminator DNA Polymerase has increased the efficiency, fidelity and range of a variety of XNAs (Pinheiro et al., 2012; Chim et al., 2017).

## Synthesis of Unnatural Base Pairs Using Therminator DNA Polymerase

In theory, new synthetic base pairs enable site-specific introduction of new chemistries in the genetic code and are at the core of efforts to create semi-synthetic organisms that can store and retrieve increased genetic information [reviewed in (Malyshev and Romesberg, 2015)]. To truly create semi-synthetic organisms, DNA polymerases must maintain high fidelity incorporation of synthetic base pairs. Currently, novel base pair structures are being paired with new generations of engineered DNA polymerases to reach this goal. To expand the genetic alphabet, synthetic base pairs have been designed and successfully and specifically incorporated by Therminator DNA Polymerase. For example, Therminator DNA Polymerase incorporates d5SICS:dMMO2 *in vitro*, however with low fidelity (Hwang and Romesberg, 2008). The next goal in the field is to increase functionalities in cells by introducing unnatural pairs into genomes and replicating with DNA polymerases such as Therminator DNA polymerase.

## DNA Sequencing and Genotyping With Therminator DNA Polymerase

### Sanger Sequencing

Sanger sequencing was the first method to sequence DNA (Sanger et al., 1977). In Sanger sequencing, a primer is extended by a DNA polymerase using a mixture of dNTPs and dye-labeled ddNTPs. After ddNTPs are incorporated, synthesis is stopped because the next nucleotide cannot be added due to the lack of the required 3′ hydroxyl group for dNMP phosphodiester bond formation. As a result, fragments labeled with different color dye-ddNTPs are produced and separated and analyzed via automated capillary electrophoresis (i.e., ABI 3730 Genetic Analyzer) [reviewed in (Slatko et al., 2011)].

Automated Sanger sequencing methods depend on engineered DNA polymerases that efficiently incorporate ddNTP terminators. The Therminator DNA Polymerase mutation was first discovered during studies focused on Vent DNA Polymerase exo- discrimination against ddNTPs for use in Sanger sequencing (Gardner and Jack, 1999). Mutating an active site alanine at position 488 to a larger amino acid (C,S,L,V, I or F) increased ddNTP incorporation by up to 15-fold compared to wild-type (Gardner and Jack, 1999). The analogous mutation in *Pyrococcus furiosus (Pfu)* (A486Y) had a similar increase in ddNTP utilization (Evans et al., 2000).

An alternative terminator, acyclonucleotides (acyNTP) substitutes a 2-hydroxyethoxymethyl group for the 2′-deoxyribofuranosyl sugar normally present in dNTP (Gardner and Jack, 2002). The increased ability to incorporate acyNTP vs. ddNTP is a distinguishing feature of hyperthermophilic archaeal DNA polymerases. DNA polymerases from *Thermococcus litoralis, Thermococcus* sp. *9*°*N7, Pyrococcus* sp. *GB-D*, and *Pfu* prefer acyNTP over ddNTPs by over 30-fold (Gardner and Jack, 2002). In addition, Therminator DNA Polymerase incorporates acyNTP 10-fold more efficiently than wild-type (Gardner et al., 2004). The catalytic incorporation efficiency of acyCTP (*k*_pol_ / *K*_D_ = 0.54) is similar to natural dCTP (*k*_pol_ / *K*_D_ = 0.72) suggesting that Therminator DNA Polymerase does not significantly discriminate between acyclonucleotides and natural nucleotides. As a result of high incorporation efficiency, dye-labeled acyNTP with Therminator DNA Polymerase were used in the LiCor/NEN Model 4200 Global IR2-automated DNA sequencer (Gardner and Jack, 2002). Despite its utility with dye-acyNTPs, Sanger sequencing technologies using Therminator DNA Polymerase remained far behind those using ThermoSequenase or TaqFS such as the ABI sequencing systems.

### Next Generation Sequencing (NGS)

As next generation sequencing (NGS) technologies evolve, new sequencing-by-synthesis (SBS) methods use dye-labeled reversible terminators in an iterative cyclic fashion (Metzker, 2010; Slatko et al., 2018). Each sequencing cycle is comprised of nucleotide incorporation, fluorescence imaging and cleavage. In the first step, an engineered DNA polymerase incorporates one fluorescently modified reversible terminator complementary to the template base. Since reversible terminators block extension of the next base, only one base is incorporated per cycle. Following incorporation and washing, imaging of the dye-terminator determines the base added. After imaging, a cleavage step removes both the terminating group and the fluorescent dye and the unblocked 3′ end is ready for the next round of incorporation, imaging and cleavage. Because wild-type DNA polymerases discriminate strongly against incorporation of nucleotides modified at the 3′-position, all SBS methods depend on engineered DNA polymerases to efficiently incorporate dye-labeled reversible terminators (Fuller et al., 2009; Chen et al., 2013; Chen, 2014).

Sequencing-by-synthesis methods use 3′-blocked reversible nucleotide terminators such as 3′-*O-*azidomethyl-dNTPs or 3′-*O*-NH-dNTPs (Hutter et al., 2010). Therminator DNA Polymerase discriminates strongly against larger modifications at the 3′ position (such as 3′-*O-*azidomethyl or 3′-*O*-NH) and incorporates these nucleotides very inefficiently with little or no detectable incorporation (Gardner et al., 2012). Therefore, researchers have introduced other mutations into the Therminator DNA Polymerase backbone to increase 3′-*O-*azidomethyl-dNTPs or 3′-*O*-NH-dNTPs incorporation efficiency and to take advantage of the underlying Therminator DNA Polymerase activities (Smith et al., 2005; Guo et al., 2008).

In contrast to 3′-modified reversible terminators, LaserGen developed a novel sequencing chemistry based on a 2-nitrobenzyl-modified 3′-OH unblocked HOMe-dNTP nucleotide that terminates synthesis after one incorporation (Litosh et al., 2011; Gardner et al., 2012). After a single incorporation, the 2-nitrobenzyl group is removed by light to reverse termination and allow the next round of synthesis to proceed. These terminators are called Lightning Terminators^TM^ and form the basis of the LaserGen/Agilent sequencing by synthesis technology. A variety of DNA polymerase incorporate Lightning Terminators but because the 3′-OH is unblocked, many DNA polymerases (such as Vent exo- or Klenow fragment exo-) continue synthesis rather than terminate (Litosh et al., 2011). Therminator DNA Polymerase has the unusual property of both incorporating Lightning Terminators and terminating synthesis despite an unblocked 3′-OH (Litosh et al., 2011). In addition, since the mismatch incorporation rate of Lightning Terminators is extremely slow, synthesis with Lightning Terminators increases incorporation fidelity compared to natural nucleotide (Gardner et al., 2012).

## Outlook for Further Therminator DNA Polymerase Engineering

Engineered DNA polymerases will continue to play key roles as molecular tools for synthesizing and copying synthetic DNA substrates and as the basis of DNA sequencing-by-synthesis techniques. To reach the potential, future DNA polymerase engineering must balance efficient incorporation of modified nucleotides with high fidelity, ideally combining high modified nucleotide incorporation efficiency with high fidelity. Currently, many of the studies described in this Review lack data on incorporation kinetics and fidelity which can provide insights into mechanisms for incorporation and discrimination. Therefore, more studies are needed to fully understand DNA polymerase mechanisms for modified nucleotide incorporation.

### Improving Modified Nucleotide Incorporation Fidelity

DNA polymerases, contain compact active sites that promote correct nucleotide binding and incorporation, and prevent misincorporation of incorrect or bulky nucleotides. Typically, DNA polymerases favor incorporation of the correct nucleotide by at least 1,000-fold (Johnson, 2010). Due to the A485L mutation, Therminator DNA Polymerase incorporates a variety of modified nucleotides but is also is prone to misincorporation of nucleotides. For example, Dunn et al. observed that the rate of correct tNTP incorporation is only approximately 10-fold faster than the incorrect tNTP (Dunn et al., 2015). Similarly, with natural nucleotides, Therminator DNA Polymerase incorporates TTP across from a correct dA in the template with a *k*_pol_ of 170 ± 4 and *K*_D_ of 73 ± 3 μM while TTP is incorporated across from an incorrect dC template is only 2-fold slower (*k*_pol_ = 70 ± 1) and has 2-fold weaker binding affinity (*K*_D_ of 150 ± 8 μM) (Gardner et al., 2012). Therefore, the Nucleotide Selectivity [(*k*_pol_/*K*_D_)_correct_/(*k*_pol_/*K*_D_)_mismatch_] for correct vs. mismatch incorporation is only 4.9-fold.

Recent advances in DNA polymerase engineering have improved the fidelity of modified nucleotide incorporation compared to Therminator DNA Polymerase. In one example, the same A485L mutation in an alternative DNA polymerase scaffold increased fidelity of amphiphilic dNTPs. Hoshino, et al. (Hoshino et al., 2016) measured fidelity by omitting a single dNamTP and testing if a DNA polymerase will halt in the absence of a correct nucleotide. In the absence of a single dNamTP, Therminator DNA Polymerase misincorporated and continued synthesis while a related DNA polymerase from *Thermococcus kodakarensis* (Tko exo-/A485L) stalled synthesis rather than misincorporate the wrong nucleotide. In addition, the Chaput group has improved incorporation fidelity of tNTP incorporation from 70 errors per 1,000 using Therminator DNA Polymerase to 4 errors per 1,000 with a related DNA polymerase KOD-RI (Tko A485R/E143A) (Horhota et al., 2005; Chim et al., 2017).

The modified nucleotide structure also influences their incorporation efficiency and fidelity. For example, Lightning Terminators are a class of reversible nucleotide terminators used in sequencing-by-synthesis methods that contain base rather than ribose modifications. Surprisingly, Lightning Terminators were incorporated with higher fidelity compared to natural nucleotides due to the 1,000-fold lower rate of incorporation of the wrong Lightning Terminator (Gardner et al., 2012). Therefore, we expect that a combination of additional DNA polymerase mutations and new nucleotide structural designs will continue to improve incorporation fidelity of modified nucleotides.

### Improving Modified Nucleotide Incorporation Efficiency

Even though many modified nucleotides can be incorporated by Therminator DNA Polymerase into short synthesis products, a continuing challenge is the synthesis of longer highly modified DNA polymers. For example, after each sequencing cycle of incorporation, imaging and unblocking during sequencing-by-synthesis methods, residual linker structures remain attached to the 3′ base. As sequencing progresses, these molecular scars accumulate and alter the structure of DNA making it a less efficient template for further DNA polymerase synthesis [(Fuller et al., 2009; Metzker, 2010; Chen et al., 2013; Chen, 2014)]. Therefore, engineered DNA polymerase that can efficiently and accurately synthesize from these highly modified DNAs are needed.

Even though the Therminator DNA Polymerase mutation (A485L) increases a variety of modified nucleotide classes, more recent studies have added additional mutations to boost incorporation efficiency and fidelity of specific modified nucleotides (Dunn et al., 2016). These secondary mutations are specifically engineered for the modified nucleotide being studied. For example, the A485L mutation combined with a second mutation (E665K) switches *Thermococcus gorgonarius* (Tgo exo-/Y409G/A485L/E665K) DNA polymerase into an engineered RNA polymerase capable of synthesizing 1.7 kb of RNA (Cozens et al., 2012). Similarly Pinheiro and colleagues identified additional mutations in the Tgo exo-/A485L that enabled efficient synthesis of XNAs (Pinheiro et al., 2012). Secondary mutations have also been introduced in Therminator DNA Polymerase to make it a more efficient DNA sequencing enzyme (Smith et al., 2005). It is likely that future engineered DNA polymerases will continue to use the A485L mutation as an important scaffold for modified nucleotide incorporation but will require additional secondary mutations that will further improve polymerase substrate specificities and fidelity.

## Author Contributions

AFG researched and wrote the paper. KJ, MB, JB, AMG, KZ, JO, and WJ researched and edited the paper. IC and VP made Figures and edited the paper.

### Conflict of Interest Statement

The authors are employed and funded by New England Biolabs, Inc., a manufacturer and vendor of molecular biology reagents, including DNA replication and repair enzymes. The funder had no role in study design, data collection and analysis, decision to publish or preparation of the manuscript and this affiliation does not affect the authors' impartiality, objectivity of data generation or its interpretation, adherence to journal standards and policies or availability of data.

## References

[B1] Anosova I. Kowal E. A. Dunn M. R. Chaput J. C. Van Horn W. D. Egli M. (2016). The structural diversity of artificial genetic polymers. Nucleic Acids Res. 44, 1007–1021. 10.1093/nar/gkv1472 26673703 PMC4756832

[B2] Baccaro A. Steck A.-L. Marx A. (2012). Barcoded nucleotides. Angew. Chem. Int. Ed. Engl. 51, 254–257. 10.1002/anie.201105717 22083884

[B3] Bergen K. Betz K. Welte W. Diederichs K. Marx A. (2013). Structures of KOD and 9 degrees N DNA polymerases complexed with primer template duplex. Chembiochem 14, 1058–1062. 10.1002/cbic.201300175 23733496

[B4] Brandis J. W. (1999). Dye structure affects Taq DNA polymerase terminator selectivity. Nucleic Acids Res. 27, 1912–1918. 10.1093/nar/27.8.1912 10101201 PMC148401

[B5] Brown J. A. Suo Z. (2011). Unlocking the sugar “steric gate” of DNA polymerases. Biochemistry 50, 1135–1142. 10.1021/bi101915z 21226515 PMC3040255

[B6] Chen C. Y. (2014). DNA polymerases drive DNA sequencing-by-synthesis technologies: both past and present. Front. Microbiol. 5:305. 10.3389/fmicb.2014.00305 25009536 PMC4068291

[B7] Chen F. Dong M. Ge M. Zhu L. Ren L. Liu G.. (2013). The history and advances of reversible terminators used in new generations of sequencing technology. Genom. Proteom. Bioinformat. 11, 34–40. 10.1016/j.gpb.2013.01.003 23414612 PMC4357665

[B8] Chen J. J. Tsai C. H. Cai X. Horhota A. T. McLaughlin L. W. Szostak J. W. (2009). Enzymatic primer-extension with glycerol-nucleoside triphosphates on DNA templates. PLoS ONE 4:e4949. 10.1371/journal.pone.0004949 19305495 PMC2654545

[B9] Chen X. Kwok P. Y. (1999). Homogeneous genotyping assays for single nucleotide polymorphisms with fluorescence resonance energy transfer detection. Genet. Anal. 14, 157–163. 10.1016/S1050-3862(98)00016-3 10084108

[B10] Chim N. Shi C. Sau S. P. Nikoomanzar A. Chaput J. C. (2017). Structural basis for TNA synthesis by an engineered TNA polymerase. Nat. Commun. 8:1810. 10.1038/s41467-017-02014-0 29180809 PMC5703726

[B11] Cozens C. Pinheiro V. B. Vaisman A. Woodgate R. Holliger P. (2012). A short adaptive path from DNA to RNA polymerases. Proc. Natl. Acad. Sci. U.S.A. 109, 8067–8072. 10.1073/pnas.1120964109 22566643 PMC3361454

[B12] Diafa S. Evéquoz D. Leumann C. J. Hollenstein M. (2017). Enzymatic synthesis of 7′,5′-Bicyclo-DNA Oligonucleotides. Chem. Asian. J. 12, 1347–1352. 10.1002/asia.201700374 28371464

[B13] Dubois C. Campbell M. A. Edwards S. L. Wengel J. Veedu R. N. (2012). Stepping towards highly flexible aptamers: enzymatic recognition studies of unlocked nucleic acid nucleotides. Chem. Commun. 48, 5503–5505. 10.1039/c2cc31316b 22540128

[B14] Dunn M. R. Larsen A. C. Zahurancik W. J. Fahmi N. E. Meyers M. Suo Z.. (2015). DNA polymerase-mediated synthesis of unbiased threose nucleic acid (TNA) polymers requires 7-deazaguanine to suppress G:G mispairing during TNA transcription. J. Am. Chem. Soc. 137, 4014–4017. 10.1021/ja511481n 25785966

[B15] Dunn M. R. Otto C. Fenton K. E. Chaput J. C. (2016). Improving polymerase activity with unnatural substrates by sampling mutations in homologous protein architectures. ACS Chem. Biol. 11, 1210–1219. 10.1021/acschembio.5b00949 26860781

[B16] Evans S. J. Fogg M. J. Mamone A. Davis M. Pearl L. H. Connolly B. A. (2000). Improving dideoxynucleotide-triphosphate utilisation by the hyper-thermophilic DNA polymerase from the archaeon *Pyrococcus furiosus*. Nucleic Acids Res. 28, 1059–1066. 10.1093/nar/28.5.1059 10666444 PMC102620

[B17] Franklin M. C. Wang J. Steitz T. A. (2001). Structure of the replicating complex of a pol alpha family DNA polymerase. Cell 105, 657–667. 10.1016/S0092-8674(01)00367-1 11389835

[B18] Fuller C. W. Middendorf L. R. Benner S. A. Church G. M. Harris T. Huang X.. (2009). The challenges of sequencing by synthesis. Nat. Biotechnol. 27, 1013–1023. 10.1038/nbt.1585 19898456

[B19] Gade C. R. Dixit M. Sharma N. K. (2016). Dideoxy nucleoside triphosphate (ddNTP) analogues: synthesis and polymerase substrate activities of pyrrolidinyl nucleoside triphosphates (prNTPs). Bioorg. Med. Chem. 24, 4016–4022. 10.1016/j.bmc.2016.06.043 27377861

[B20] Gardner A. Jack W. (2002). Acyclic and dideoxy terminator preferences denote divergent sugar recognition by archaeon and Taq DNA polymerases. Nucleic Acids Res. 30, 605–613. 10.1093/nar/30.2.605 11788725 PMC99817

[B21] Gardner A. F. Jack W. E. (1999). Determinants of nucleotide sugar recognition in an archaeon DNA polymerase. Nucleic Acids Res. 27, 2545–2553. 10.1093/nar/27.12.2545 10352184 PMC148459

[B22] Gardner A. F. Joyce C. M. Jack W. E. (2004). Comparative kinetics of nucleotide analog incorporation by Vent DNA polymerase. J. Biol. Chem. 279, 11834–11842. 10.1074/jbc.M308286200 14699133

[B23] Gardner A. F. Wang J. Wu W. Karouby J. Li H. Stupi B. P.. (2012). Rapid incorporation kinetics and improved fidelity of a novel class of 3′-OH unblocked reversible terminators. Nucleic Acids Res. 40, 7404–7415. 10.1093/nar/gks330 22570423 PMC3424534

[B24] Giraut A. Abu El-Asrar R. Marlière P. Delarue M. Herdewijn P. (2012). 2′-Deoxyribonucleoside phosphoramidate triphosphate analogues as alternative substrates for *E. coli* polymerase III. Chembiochem 13, 2439–2444. 10.1002/cbic.201200413. 23023962

[B25] Guo J. Xu N. Li Z. Zhang S. Wu J. Kim D. H.. (2008). Four-color DNA sequencing with 3′-O-modified nucleotide reversible terminators and chemically cleavable fluorescent dideoxynucleotides. Proc. Natl. Acad. Sci. U.S.A. 105, 9145–9150. 10.1073/pnas.0804023105 18591653 PMC2442126

[B26] Hashimoto H. Nishioka M. Fujiwara S. Takagi M. Imanaka T. Inoue T.. (2001). Crystal structure of DNA polymerase from hyperthermophilic archaeon *Pyrococcus kodakaraensis* KOD1. J. Mol. Biol. 306, 469–477. 10.1006/jmbi.2000.4403 11178906

[B27] Holzberger B. Marx A. (2009). Enzymatic synthesis of perfluoroalkylated DNA. Bioorg. Med. Chem. 17, 3653–3658. 10.1016/j.bmc.2009.03.063 19401268

[B28] Holzberger B. Strohmeier J. Siegmund V. Diederichsen U. Marx A. (2012). Enzymatic synthesis of 8-vinyl- and 8-styryl-2′-deoxyguanosine modified DNA–novel fluorescent molecular probes. Bioorg. Med. Chem. Lett. 22, 3136–3139. 22483394 10.1016/j.bmcl.2012.03.056

[B29] Horhota A. Zou K. Ichida J. K. Yu B. McLaughlin L. W. Szostak J. W.. (2005). Kinetic analysis of an efficient DNA-dependent TNA polymerase. J. Am. Chem. Soc. 127, 7427–7434. 10.1021/ja0428255 15898792 PMC5042361

[B30] Hoshino H. Kasahara Y. Fujita H. Kuwahara M. Morihiro K. Tsunoda S. I.. (2016). Consecutive incorporation of functionalized nucleotides with amphiphilic side chains by novel KOD polymerase mutant. Bioorg. Med. Chem. Lett. 26, 530–533. 10.1016/j.bmcl.2015.11.079 26627581

[B31] Houlihan G. Arangundy-Franklin S. Holliger P. (2017a). Engineering and application of polymerases for synthetic genetics. Curr. Opin. Biotechnol. 48, 168–179. 10.1016/j.copbio.2017.04.004 28601700

[B32] Houlihan G. Arangundy-Franklin S. Holliger P. (2017b). Exploring the chemistry of genetic information storage and propagation through polymerase engineering. ACC. Chem. Res. 50, 1079–1087. 10.1021/acs.accounts.7b00056 28383245 PMC5406124

[B33] Hutter D. Kim M.-J. Karalkar N. Leal N. A. Chen F. Guggenheim E.. (2010). Labeled nucleoside triphosphates with reversibly terminating aminoalkoxyl groups. Nucl. Nucleot. Nucleic Acids 29, 879–895. 10.1080/15257770.2010.536191 21128174 PMC3858015

[B34] Hwang G. T. Romesberg F. E. (2008). Unnatural substrate repertoire of A, B, and X family DNA polymerases. J. Am. Chem. Soc. 130, 14872–14882. 10.1021/ja803833h 18847263 PMC2675700

[B35] Ichida J. K. Horhota A. Zou K. McLaughlin L. W. Szostak J. W. (2005a). High fidelity TNA synthesis by Therminator polymerase. Nucleic Acids Res. 33, 5219–5225. 10.1093/nar/gki840 16157867 PMC1214552

[B36] Ichida J. K. Zou K. Horhota A. Yu B. McLaughlin L. W. Szostak J. W. (2005b). An *in vitro* selection system for TNA. J. Am. Chem. Soc. 127, 2802–2803. 10.1021/ja045364w 15740086 PMC5072288

[B37] Johnson K. A. (2010). The kinetic and chemical mechanism of high-fidelity DNA polymerases. Biochim. Biophys. Acta. 1804, 1041–1048. 10.1016/j.bbapap.2010.01.006 20079883 PMC3047511

[B38] Kasuya T. Hori S. Hiramatsu H. Yanagimoto T. (2014). Highly accurate synthesis of the fully 2′-fluoro-modified oligonucleotide by Therminator DNA polymerases. Bioorg. Med. Chem. Lett. 24, 2134–2136. 10.1016/j.bmcl.2014.03.035 24703229

[B39] Kataoka M. Kouda Y. Sato K. Minakawa N. Matsuda A. (2011). Highly efficient enzymatic synthesis of 3′-deoxyapionucleic acid (apioNA) having the four natural nucleobases. Chem. Commun. 47, 8700–8702. 10.1039/C1CC12980E 21725575

[B40] Kataoka M. Sato K. Matsuda A. (2008). Synthesis of 3′-deoxyapionucleoside triphosphates and their incorporation into DNA by DNA polymerase. Nucleic Acids Symp. Ser. 52, 281–282. 10.1093/nass/nrn142 18776363

[B41] Kong H. Kucera R. B. Jack W. E. (1993). Characterization of a DNA polymerase from the hyperthermophile archaea *Thermococcus litoralis*. Vent DNA polymerase, steady state kinetics, thermal stability, processivity, strand displacement, and exonuclease activities. J. Biol. Chem. 268, 1965–1975. 8420970

[B42] Kropp H. M. Betz K. Wirth J. Diederichs K. Marx A. (2017). Crystal structures of ternary complexes of archaeal B-family DNA polymerases. PLoS ONE, 12:e0188005. 10.1371/journal.pone.0188005 29211756 PMC5718519

[B43] Lapa S. A. Chudinov A. V. Timofeev E. N. (2016). The toolbox for modified aptamers. Mol. Biotechnol. 58, 79–92. 10.1007/s12033-015-9907-9 26607475

[B44] Litosh V. A. Wu W. Stupi B. P. Wang J. Morris S. E. Hersh M. N.. (2011). Improved nucleotide selectivity and termination of 3′-OH unblocked reversible terminators by molecular tuning of 2-nitrobenzyl alkylated HOMedU triphosphates. Nucleic Acids Res. 39:e39. 10.1093/nar/gkq1293 21227920 PMC3064798

[B45] Malyshev D. A. Romesberg F. E. (2015). The expanded genetic alphabet. Angew. Chem. Int. Ed. Engl. 54, 11930–11944. 10.1002/anie.201502890 26304162 PMC4798003

[B46] Matyašovsk,ý J. Pohl R. Hocek M. (2018). 2-allyl- and propargylamino-dATPs for site-specific enzymatic introduction of a single modification in the minor groove of DNA. Chemistry 24, 14938–14941. 10.1002/chem.201803973 30074286 PMC6221035

[B47] Metzker M. L. (2010). Sequencing technologies - the next generation. Nat. Rev. Genet. 11, 31–46. 10.1038/nrg2626 19997069

[B48] Nawale G. N. Gore K. R. Höbartner C. Pradeepkumar P. I. (2012). Incorporation of 4′-C-aminomethyl-2′-O-methylthymidine into DNA by thermophilic DNA polymerases. Chem. Commun. 48, 9619–9621. 10.1039/C2CC35222B 22908130

[B49] Ogino T. Sato K. Matsuda A. (2010). Incorporation of 2′-deoxy-2′-isonucleoside 5′-triphosphates (iNTPs) into DNA by A- and B-family DNA polymerases with different recognition mechanisms. Chembiochem 11, 2597–2605. 10.1002/cbic.201000449 21108267

[B50] Pinheiro V. B. Taylor A. I. Cozens C. Abramov M. Renders M. Zhang S.. (2012). Synthetic genetic polymers capable of heredity and evolution. Science 336, 341–344. 10.1126/science.1217622 22517858 PMC3362463

[B51] Prober J. M. Trainor G. L. Dam R. J. Hobbs F. W. Robertson C. W. Zagursky R. J.. (1987). A system for rapid DNA sequencing with fluorescent chain-terminating dideoxynucleotides. Science 238, 336–341. 10.1126/science.2443975 2443975

[B52] Renders M. Emmerechts G. Rozenski J. Krecmerová M. Holý A. Herdewijn P. (2007). Enzymatic synthesis of phosphonomethyl oligonucleotides by Therminator polymerase. Angew. Chem. Int. Ed. Engl. 46, 2501–2504. 10.1002/anie.200603435 17310479

[B53] Renders M. Lievrouw R. Krecmerová M. Holý A. Herdewijn P. (2008). Enzymatic polymerization of phosphonate nucleosides. Chembiochem 9, 2883–2888. 10.1002/cbic.200800494 19006151

[B54] Rodriguez A. C. Park H. W. Mao C. Beese L. S. (2000). Crystal structure of a pol alpha family DNA polymerase from the hyperthermophilic archaeon *Thermococcus* sp. 9 degrees N-7. J. Mol. Biol. 299, 447–462. 10.1006/jmbi.2000.3728 10860752

[B55] Sanger F. Nicklen S. Coulson A. R. (1977). DNA sequencing with chain-terminating inhibitors. Proc. Natl. Acad. Sci. U.S.A. 74, 5463–5467. 10.1073/pnas.74.12.5463 271968 PMC431765

[B56] Schmidt M. (2010). Xenobiology: a new form of life as the ultimate biosafety tool. Bioessays 32, 322–331. 10.1002/bies.200900147 20217844 PMC2909387

[B57] Sievers F. Higgins D. G. (2018). Clustal omega for making accurate alignments of many protein sequences. Protein. Sci. 27, 135–145. 10.1002/pro.3290 28884485 PMC5734385

[B58] Slatko B. E. Gardner A. F. Ausubel F. M. (2018). Overview of next-generation sequencing technologies. Curr. Protoc. Mol. Biol. 122:e59. 10.1002/cpmb.59 29851291 PMC6020069

[B59] Slatko B. E. Kieleczawa J. Ju J. Gardner A. F. Hendrickson C. L. Ausubel F. M. (2011). “First generation” automated DNA sequencing technology. Curr. Protoc. Mol. Biol. Chapter 7, Unit7 2. 10.1002/0471142727.mb0702s96 21987057

[B60] Smith G. P. Bailey D. M. Sanches R. M. Swerdlow H. Earnshaw D. J. (2005). Modified Polymerases for Improved Incorporation of Nucleotide Analogues. World Intellectual Property Organization, WO/2005/024010.

[B61] Southworth M. W. Kong H. Kucera R. B. Ware J. Jannasch H. W. Perler F. B. (1996). Cloning of thermostable DNA polymerasesfrom hyperthermophilic marine Archaea with emphasis on *Thermococcus* sp. 9 degrees N-7 and mutations affecting 3′-5′ exonuclease activity. Proc. Natl. Acad. Sci. U.S.A. 93, 5281–5285. 10.1073/pnas.93.11.5281 8643567 PMC39236

[B62] Steitz T. A. (1999). DNA polymerases: structural diversity and common mechanisms. J. Biol. Chem. 274, 17395–17398. 10.1074/jbc.274.25.17395 10364165

[B63] Uemori T. Ishino Y. Toh H. Asada K. Kato I. (1993). Organization and nucleotide sequence of the DNA polymerase gene from the archaeon *Pyrococcus furiosus*. Nucleic Acids Res. 21, 259–265. 10.1093/nar/21.2.259 8441634 PMC309101

[B64] Vester B. Wengel J. (2004). LNA (locked nucleic acid): high-affinity targeting of complementary RNA and DNA. Biochemistry 43, 13233–13241. 10.1021/bi0485732 15491130

[B65] Williams J. G. K. Steffens D. L. Anderson J. P. Urlacher T. M. Lamb D. T. Grone D. L.. (2008). An artificial processivity clamp made with streptavidin facilitates oriented attachment of polymerase-DNA complexes to surfaces. Nucleic Acids Res. 36:e121. 10.1093/nar/gkn531 18723573 PMC2566871

[B66] Williams J. S. Kunkel T. A. (2014). Ribonucleotides in DNA: origins, repair and consequences. DNA Repair. 19, 27–37. 10.1016/j.dnarep.2014.03.029 24794402 PMC4065383

[B67] Xia S. Konigsberg W. H. (2014). RB69 DNA polymerase structure, kinetics, and fidelity. Biochemistry 53, 2752–2767. 10.1021/bi4014215 24720884 PMC4018061

[B68] Xiao M. Phong A. Ha C. Chan T. F. Cai D. Leung L.. (2007). Rapid DNA mapping by fluorescent single molecule detection. Nucleic Acids Res. 35:e16. 10.1093/nar/gkl1044 17175538 PMC1807959

[B69] Xiao Y. Liu Q. Tang X. Yang Z. Wu L. He Y. (2017). Mirror-image thymidine discriminates against incorporation of deoxyribonucleotide triphosphate into DNA and repairs itself by DNA polymerases. Bioconjug. Chem. 28, 2125–2134. 10.1021/acs.bioconjchem.7b00301 28686433

[B70] Yang S. Herdewijn P. (2011). Polymerase-dependent DNA synthesis from phosphoramidate-activated nucleotides. Nucl. Nucleot. Nucleic Acids. 30, 597–608. 10.1080/15257770.2011.598491 21888550

[B71] Yu H. Zhang S. Dunn M. R. Chaput J. C. (2013). An efficient and faithful *in vitro* replication system for threose nucleic acid. J. Am. Chem. Soc. 135, 3583–3591. 10.1021/ja3118703 23432469

[B72] Zhang S. Chaput J. C. (2013). Synthesis and enzymatic incorporation of alpha-L-threofuranosyl adenine triphosphate (tATP). Bioorg. Med. Chem. Lett. 23, 1447–1449. 10.1016/j.bmcl.2012.12.080 23352269

[B73] Zhang S. Yu H. Chaput J. C. (2013). Synthesis of threose nucleic acid (TNA) triphosphates and oligonucleotides by polymerase-mediated primer extension. Curr. Protoc. Nucleic Acid. Chem. Chapter 4, Unit 4.54. 10.1002/0471142700.nc0454s52 23512696

